# The ‘Matthew effect’ in Chinese kindergarten principals’ professional development: a mixed-methods study

**DOI:** 10.3389/fpsyg.2023.1118787

**Published:** 2023-06-07

**Authors:** Yu Qian, Shiyuan Zhangchen, Hui Li

**Affiliations:** ^1^Department of Early Childhood Education, Faculty of Education, East China Normal University, Shanghai, China; ^2^Shanghai Institute of Early Childhood Education, Shanghai Normal University, Shanghai, China

**Keywords:** kindergarten principals, professional development needs, early childhood education, Matthew effect, principal training

## Abstract

Principals play a leading role in kindergarten quality improvement, and thus their needs for professional development (PD) should be understood and met. This national study adopted a mixed-methods approach to survey 3,065 kindergarten principals in China and interviewed 16 of them. First, the latent profile analysis of survey data yielded three profiles of PD needs: (1) low (7.5%), (2) medium (22.2%), and (3) high profiles (70.3%), indicating 70.3% of Chinese principals need PD badly. The high-profile group features ‘inexperienced principals working at newly established private kindergartens’, the most disadvantaged among the three groups. Second, ANOVA tests revealed significant rural–urban and public-private differences in Chinese principals’ professional backgrounds and PD needs. In particular, significant public-private and rural–urban differences were observed in the principals’ ‘current degree’ (*F*s > 63, *ps* < 0.001) and ‘desired degree’ (*F*s > 39, *ps* < 0.001). The rural principals aspired more than their urban counterparts to obtain ‘a higher degree” or ‘a certificate’ (*ps* < 0.05). Third, the follow-up interviews confirmed remarkable rural–urban and public-private gaps in PD needs, indicating a noticeable ‘Matthew effect’: the poor got less, whereas the rich got more. The implications for future PD policy and program development are discussed.

## 1. Introduction

The ‘Matthew effect’ refers to a pattern in which those who begin with advantages accumulate more over time, while those who begin with disadvantages become more disadvantaged over time (Merton, 1968, 1973; Dannefer, 1987). This effect is extensively evident in Chinese early childhood education (ECE). For example, in China, public kindergartens (catering to young children ages 3–6) tend to have better educational quality and resources than private kindergartens. This is because the local governments only fund public kindergartens and leave those private ones fending for themselves, like ‘Cinderella’ crying in the kitchen. In addition, rural kindergartens often receive lower levels of funding than urban ones. These rural–urban and public-private gaps have further exacerbated the ‘Matthew effect,’ causing more significant educational inequalities and the associated ‘3A’ problems (Li et al., 2017): ‘accessibility’ (more difficulty enrolling in public kindergartens in rural areas), ‘affordability’ (expensive tuition in private kindergartens), and ‘accountability’ (worse quality in rural and private kindergartens). Since 2010, the Chinese government has drastically increased its input into teacher professional development (PD) to enhance the quality of teachers and principals. However, the financial input and professional resources went primarily to public kindergartens in urban areas, leaving those private rural ones unattended. The private-public dichotomy, urban–rural divide, and associated prejudices might have caused a ‘Matthew effect’ in early childhood teacher education. However, no empirical evidence has confirmed this phenomenon in the field of teacher PD, especially among kindergarten principals who play a leading role in kindergarten quality improvement (Rodd, 2001; Bloom, 2004; Bloom and Bella, 2005). To fill this gap, this study adopted a mixed-methods approach to explore the ‘Matthew effect’ in Chinese kindergarten principals’ PD needs.

## 2. Literature review

### 2.1. The private-public and rural–urban gaps and Matthew effect in ECE

In 1903, the first Chinese public kindergarten was established in Wuhan. Since then, rural–urban and public-private ECE gaps have emerged and persisted. Existing studies have extensively reported these gaps in enrolment ratios, tuition fees, teacher-child ratios, and teachers’ qualifications (Li et al., 2019; Qian, 2019; Qian et al., 2022). First, the enrolment rate in rural kindergartens was much lower than those in urban kindergartens. For example, in most metropolitan areas, enrolment in ECE was almost 100%, nearly 30% higher than that in some western rural regions (Qian, 2019). Second, private kindergartens’ tuition fees are higher because they usually have fewer resources than public ones (Xie and Li, 2020). Third, the teacher-child ratio in rural areas was lower than in urban areas. For example, the teacher-child ratio was approximately 1:25 in Jiangsu, Chongqing, and Sichuan rural kindergartens, compared with 1:15 in most urban kindergartens (Gov. CHN-Department of Development and Planning of Chinese Ministry of Education, 2021). Fourth, the teachers’ qualifications in rural or private kindergartens were lower than those in urban and public kindergartens (Luo, 2021; Yin, 2022).

As mentioned above, Li et al. (2017) described how rural–urban and public-private ECE gaps have caused the ‘3A’ problems of accessibility, affordability, and accountability. These 3A problems in ECE will likely cause inequality in children’s abilities, achievements, health, and professional success in adulthood. Moreover, as the Matthew Effect indicated, urban or public kindergartens that begin with advantages accumulate more over time, while those rural or private schools that start with disadvantages become more disadvantaged over time. To solve these problems, the central government released two critical documents, marking a new era for ECE services in China in 2010. The first was the *National Medium to Long Term Planning Outline for Educational Reform and Development* (the *Outline*), which stated three goals: (1) promoting the universal provision of ECE to provide fair opportunities; (2) increasing government responsibilities in developing public kindergartens and supporting private schools; and (3) facilitating rural kindergartens. The second document was the *State Council’s Several Opinions on the Current Development of Early Childhood Education,* which prompted governments at all levels to strengthen PD for kindergarten teachers. Since then, ECE services in China have witnessed a ‘Great Leap Forward’ (Li et al., 2017).

According to the Chinese Ministry of Education’s (MOE) *Statistical Bulletin 2021*, there are 48,182,600 children attending 291,700 kindergartens (123,700 of which are public and 168,000 are private). Approximately half of all children (50.63%) are enrolled in public kindergartens (49.37% are in private services). Furthermore, 128,650 (41.72%) are in urban kindergartens, and 179,370 (58.28%) are in rural areas. However, even though the enrolment rate has almost doubled over the past decade (by 2021, 88.1% of children nationwide were enrolled, compared with 50.9% in 2009), the rural–urban and private-public gaps remain an issue. Recent studies reported that the quality of rural and private kindergartens tends to lag behind that of urban and public schools (Zhang et al., 2020; Jiang and Liu, 2022). Furthermore, significant rural–urban and private-public gaps were identified in child outcomes regarding social skills, basic knowledge, language competence, and overall development (Zhang et al., 2020).

Therefore, as part of efforts to narrow the gaps between rural–urban and public-private kindergartens, the Chinese government has emphasized the importance of kindergarten principals’ PD. In 2015, the educational authorities of China released the *Professional Standards for Kindergarten Principals* (the *“Principals’ Standards”* hereafter) to promote kindergarten principals’ PD. This document provides an ‘important basis for formulating principals’ qualification, PD program, and assessment standards’. Accordingly, many PD programs were sponsored and delivered by local and central governments, training institutes, and universities.

### 2.2. Professional development needs in Chinese kindergarten principals

Principals are responsible for a kindergarten’s daily operations, supervising teaching staff, directing program planning, and administering overall performance (Rodd, 2001; Bloom, 2004; Bloom and Bella, 2005; Jiao and Liu, 2022). Currently, China has 308,380 kindergarten principals (some kindergartens have more than one principal); half are working in private kindergartens and half in rural areas. Their leadership is critical or decisive to school development and quality improvement (Sims et al., 2015). This is because they can create a climate that promotes children’s optimal growth and development and implement effective educational systems (Kagan and Bowman, 1997; Bloom, 2004). In addition, several studies have indicated that effective leaders could improve service quality (Bloom and Sheerer, 1992; Rodd, 1997; Brownlee et al., 2009; Day and Sammons, 2016; Al-Hamad et al., 2020). As Grogin and Andrews (2002) claimed, ‘the school principal is a key lynchpin between teacher development and school improvement’ (p. 249).

However, most Chinese kindergarten principals are selected from the teacher team; thus, they often lack proper preparation and face a huge transition in their roles (Chen, 2022). As the national *Kindergarten Working Regulation (2016)* states, the kindergarten principal must have a teaching certificate, a college degree or above, more than three years of working experience in kindergarten, a certain amount of leadership, and a certificate for the principal’s training. In previous studies, few principals stated they were well-prepared; most described the transition into their new administrative role as overwhelming (Wu, 2021; Jiao and Liu, 2022). Therefore, they need specialized and continuous PD to develop their expertise, skills, and leadership (Scott, 1999; Burchinal et al., 2002; Grogin and Andrews, 2002; Darling-Hammond et al., 2017).

There is no doubt among policymakers and practitioners about the importance of PD, and most experts agree that needs analysis is critical to successful PD (Kirkpatrick and Kirkpatrick, 2007; Ng and Szeto, 2016). In other societies, many studies have investigated principals’ PD needs (Norton and Abramowitz, 1981; Whitebook et al., 2011; Hallinger and Chen, 2015). However, few have examined the PD needs of kindergarten principals in China (Wang and Jiao, 2015; Jiao and Liu, 2022). Moreover, none have explored the private-public and rural–urban gaps in PD needs among Chinese principals. The existing studies confirmed that Chinese principals had inadequate PD opportunities. Their PD needs have been ignored (Wang and Jiao, 2015; Jiao and Liu, 2022), particularly among those private kindergartens in central and western rural areas (Wu, 2021; Qu, 2022).

### 2.3. The current study

Aiming to build a high-quality and professional principal team, the MOE launched the national Kindergarten Principal PD program in 2014. In particular, the authorities launched a national Key PD program targeting the most experienced and effective public school principals, hoping to ‘exert their radiation effect’ on principals’ PD (Jiao and Liu, 2022). The national Key Principal program annually provides a one-month free PD program to the eight most-experienced public principals selected from each province. However, this national PD program followed the existing public-private dichotomy; thus, the rural–urban divide might have exacerbated the educational inequalities between the rich and poor. This means that public kindergartens in urban areas would get more and better PD resources than those private ones in rural areas. Therefore, one would naturally ask questions such as “are there any rural–urban and private-public gaps in Chinese principals’ PD needs” and “is there a Matthew effect in PD or not?” The answers to these questions would help policymakers review the national PD program, improve the policies and practices, and eventually facilitate Chinese principals’ PD. To achieve this objective, this national survey study adopted a mixed-methods approach to explore the inequalities in PD needs empirically. The following questions guided this study:

1. Are there significant rural–urban differences in PD needs among Chinese kindergarten principals?
2. Are there significant private-public differences in PD needs among Chinese kindergarten principals?
3. Are there any latent profiles of PD needs? And who needs PD the most?

## 3. Methods

### 3.1. Participants

This is a quantitative-qualitative sequential mixed-methods study, as we first conducted a national survey study and then an in-depth interview study. Classified random sampling was conducted for the national survey study. There are remarkable rural–urban and east–west gaps in social and economic development in China. The economy in eastern China is more advanced than in western and central China. To understand the real situation of PD in varying areas of China, we included 10 regions representing different parts of China. First, we randomly selected five regions representing eastern China (Beijing, Shanghai, Jiangsu, Zhejiang, Fujian) and five representing central and western China (Neimenggu, Chongqing, Xinjiang, Heilongjiang, Sichuan). Second, between July and December 2021, local teaching/research staff helped to disseminate anonymous online questionnaires to regional principals’ WeChat groups (WeChat is the largest online communication app in China). Third, the kindergarten principals were invited to complete an online informed consent form, after which they received an online questionnaire via WeChat at www.wjx.cn. Finally, 3,065 participants completed the survey after answering all the questions, and the percentage of valid questionnaires was 96.67% (*n* = 2,963). This comprised 1,949 public and 1,014 private kindergarten principals; 1,751 worked in urban and 1,212 in rural areas. In total, 1,068 were urban public principals, 881 worked in rural public schools, 683 worked in urban private schools, and 331 worked in rural private schools. More than half (53.29%) were under 40 years old, and 84% of the participants had a degree majored in ECE. One-fifth worked in new kindergartens founded less than three years ago. Full demographic information is presented in Table 1.

**Table 1:** Participants’ demographic information (*n* = 2,963).

	Description	Participants	Percentage
Area	Eastern	1,651	55.72%
	Central and western	1,312	44.28%
Age	20–30	356	12.01%
	31–40	1,223	41.28%
	41–50	1,157	39.05%
	51 or older	227	7.66%
Years of work experience	3 years or less	131	4.42%
	4–7 years	377	12.72%
	8–10 years	359	12.12%
	11–15 years	561	18.93%
	More than 15 years	1,535	51.81%
Years of work experience as principal	1–3 years	1,160	39.15%
	4–7 years	726	24.5%
	8–10 years	436	14.71%
	11–15 years	308	10.39%
	More than 15 years	333	11.24%
Major	ECE	2,490	84.04%
	Education	274	9.25%
	Not education-related	189	6.72%
Current educational level	Master’s degree	95	3.21%
	Bachelor’s degree	2,248	75.87%
	College degree	560	18.9%
	Teacher training school	47	1.59%
	High school	13	0.44%
Years since school established	1–3 years	567	19.14%
	4–7 years	558	18.83%
	8–12 years	565	19.07%
	13–20 years	482	16.27%
	21 years or more	791	26.7%
School type	Urban public school	1,068	36.04%
	Rural public school	881	29.73%
	Urban private school	683	23.05%
	Rural public school	331	11.17%
Urban–rural	Urban	1751	59.1%
	Rural	1,212	40.9%
Public-private	Public	1,949	65.78%
	Private	1,014	34.22%
Total		2,963	100%

In the follow-up interview study, 16 participants were sampled from the survey study to better understand the factors underlying obstacles to their PD. Four participants were randomly sampled from each group: urban public, urban private, rural public, and rural private schools. In addition, the 16 participants were invited to complete individual semi-structured interviews. After obtaining their informed consent, the interviews were conducted between January and April 2022; interviews usually lasted between 30 and 60 min and were conducted via WeChat. Their demographic characteristics are presented in Table 2.

**Table 2:** Demographic characteristics of interviewees (*n* = 16).

Code	Category	Gender	Degree	Years in administrative position
A	Urban public kindergarten principals	Female	Bachelor’s	2
B	Urban public kindergarten principals	Female	Master’s	5
C	Urban public kindergarten principals	Female	Bachelor’s	3
D	Urban public kindergarten principals	Female	College	3
E	Urban private kindergarten principals	Male	Master’s	7
F	Urban private kindergarten principals	Female	College	3
G	Urban private kindergarten principals	Female	College	2
H	Urban private kindergarten principals	Female	Bachelor’s	12
I	Rural public kindergarten principals	Female	College	5
J	Rural public kindergarten principals	Female	College	3
K	Rural public kindergarten principals	Female	College	6
L	Rural public kindergarten principals	Female	Bachelor’s	9
M	Rural private kindergarten principals	Female	College	2
N	Rural private kindergarten principals	Female	College	4
O	Rural private kindergarten principals	Female	College	3
P	Rural private kindergarten principals	Female	High school	18

This study was approved by East China Normal University Ethics Review Committee (HR004-2021). All data containing personal information were anonymized or stored securely to protect participants’ privacy.

### 3.2. Measures

#### 3.2.1. Principals’ PD needs and obstacles questionnaire

This questionnaire investigated kindergarten principals’ backgrounds, PD needs, and obstacles influencing their PD. It was developed and reviewed by five experts in ECE. Among them, three were university researchers in ECE, and two were practice experts; all were familiar with the principal’s PD. In addition, a pilot study was conducted in a non-participating region to ensure the contents of the questionnaires were valid, clear, and easy to understand, and the completion time was reasonable. Subsequently, necessary clarifications and corresponding changes were made to refine and finalize the instruments.

The final version of the questionnaire consisted of two major parts. Part one included 11 items relating to participants’ background information, including their current educational level and the level they desired to attain through future PD programs. Part two contained 18 items scored on 5-point Likert scales ranging from 1 (*strongly disagree*) to 5 (*strongly agree*). These items assessed kindergarten principals’ PD needs and obstacles from three perspectives. First, PD needs were evaluated using eight items; examples include: ‘My PD need is to improve my marketing skills and ‘My PD need is to obtain a higher degree’. Second, preferred PD program content contained six items designed according to the six domains defined by the *Principals’ Standards*: ‘Planning the development of kindergarten’, ‘Creating educational culture’, ‘Leading care and education’, ‘Guiding teachers’ development’, ‘Improving management’, and ‘Adapting to the environment’. Finally, obstacles to PD contained four items; examples include: ‘The main obstacle to my PD is that the PD program contents were too theoretical’ and ‘The main obstacle to my PD is the inconvenient location of the PD program’.

The overall Cronbach’s α coefficient for this questionnaire was 0.918. Fitness tests showed good validity: KMO = 0.922; significance probability = 0.000. A three-factor model (e.g., PD needs, preferred PD program content, and obstacles to PD) was generated for the scale using the principal-axis factoring of the Direct Oblimin method, which could explain 64.052% of the total variance, implying that the construct validity of the scale was acceptable. The eigenvalues of the three constructs are 5.052, 3.016, and 1.396, respectively. We conducted confirmatory factor analysis using 2,963 formally collected questionnaires to verify the factor structure of each dimension of the scale. The scale demonstrated an acceptable model fit, χ2 = 3811.919, df = 132, *p* < 0.001, comparative fit index (CFI) = 0.887, goodness-of-fit index (GFI) = 0.884, root of the mean square residual (RMR) = 0.06, and root mean square error of approximation (RMSEA) = 0.097. For the CFI and GFI, their values are between 0–1, with closer to 0 indicating a worse fit and closer to 1 indicating a better fit. RMSEA values less than 0.10 suggest an acceptable model fit, and RMR values of 0.08 or less than 0.1 indicate an acceptable fit.

#### 3.2.2. Semi-structured interviews

Li et al. (2017) proposed the aforementioned 3A framework for ECE policy study (accessibility, affordability, accountability); it provides a reliable, comparable, appropriate, and consistent measure to assess progress in ECE policies in Asia-Pacific countries. In interviews with the 16 selected participants, we extended the framework to explore 3A problems associated with principals’ PD needs based on questions such as (1) Accessibility: Is it difficult for you to access PD opportunities? (2) Affordability: Do you have adequate time and funds for PD? (3) Accountability: Was your PD program effective? Are there any relevant policies or extra funds that could improve the quality? Participants were also asked to provide further comments on the accessibility, affordability, and accountability of their PD. Following this, open-ended interview questions were posed, focusing on their PD needs. The interview protocol was developed to understand participants’ views on conflicts and the major obstacles to their PD. All the interviews were conducted and audio-recorded in Mandarin Chinese, with informed consent obtained from all participants. Each interview lasted between 30 min and 1 h, depending on their availability. All interviews were transcribed into a Word document and then subjected to discourse analysis.

### 3.3. Data analysis

#### 3.3.1. Quantitative data analysis

From the 3,065 completed questionnaires, 102 were deleted for the following reasons: (1) participants completed the questionnaire in less than 60 s, or (2) over 90% of the responses to scaled questions were identical. Thus, 2,963 valid questionnaires were included in the final dataset (the percentage of valid questionnaires was 96.7%). Second, we conducted two-way ANOVA tests to compare between- and within-group means (i.e., public vs. private; urban vs. rural) using SPSS 26.0. Pairwise comparison differences were considered significant at *p* < 0.05. We also employed latent profile analysis (LPA) to identify the PD needs patterns of different participants using Mplus 8.3. LPA identifies latent subpopulations within a population based on a certain set of variables (Spurk et al., 2020). It is helpful for us to identify the subgroups with different PD needs within large, heterogeneous populations in China.

#### 3.3.2. Qualitative data analysis

We used two techniques to ensure the trustworthiness of the qualitative data analysis, including peer debriefing and inquiry auditing. First, in the peer debriefing stage, the first author verified that the three themes could accurately represent patterned responses and the meaning of the interview data. Second, t*he first and second authors analyzed the interview data.* Third, the last author, a professor and senior ECE researcher, acted as an inquiry auditor to ensure that qualitative data collection and analysis processes were sufficiently rigorous.

## 4. Results

### 4.1. Demographic information of participants

**Figure 1:**
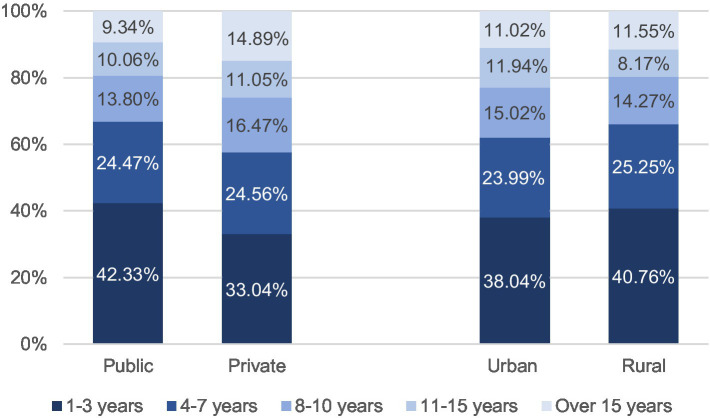
Years of administrative experience among rural, urban, public, and private principals.

We used descriptive statistics (i.e., means and SDs) to summarize the demographic data of all 2,963 participants. Significant differences were found in the years of administrative experience, current degrees, and desired degrees. Most participants (*n* = 1,886, 63.65%) indicated that they had worked as principals for less than 7 years, while 1,160 (39.15%) reported administrative work experience of fewer than 3 years. Only 333 (11.24%) principals had more than 15 years of administrative experience. Compared with private (57.6%) and urban (62.03%) participants, more public (66.8%) and rural (66.01%) principals had less than 7 years of administrative experience. ANOVA tests showed no significant difference in administrative experience between rural–urban participants (*F* = 3.389, *p* = 0.066). However, significant statistical differences were found between public and private participants (*F* = 33.650, *p* < 0.001). As Figure 1 shows, the proportion of public principals with less than 3 years of administrative experience was nearly 10% higher than that of private principals (public = 42.33%; private = 33.04%).

More than three-quarters of respondents (75.87%) reported having a bachelor’s degree. Public school participants in eastern China had the highest level of bachelor’s degrees (92.77%), while those in rural private kindergartens had the lowest bachelor’s degrees (41.09%). There were significant differences between rural–urban and public-private participants’ current degrees (rural–urban: *χ*^2^ = 79.723, *p* < 0.001; public-private: *χ*^2^ = 465.897, *p* < 0.001) and desired degrees (rural–urban: *χ*^2^ = 42.422, *p* < 0.001; public-private: *χ*^2^ = 135.209, *p* < 0.001).

Overall, urban participants had a higher education level than rural participants. More urban participants had bachelor’s degrees (77.33%) than rural ones (73.76%). Nearly 5% of urban principals had a master’s degree, compared with 0.66% of rural principals. In addition, most urban participants desired a master’s degree (urban = 67.85%; rural = 58.09%), while more rural principals desired a bachelor’s degree through future PD (urban = 31.07%; rural = 38.45%).

**Figure 2:**
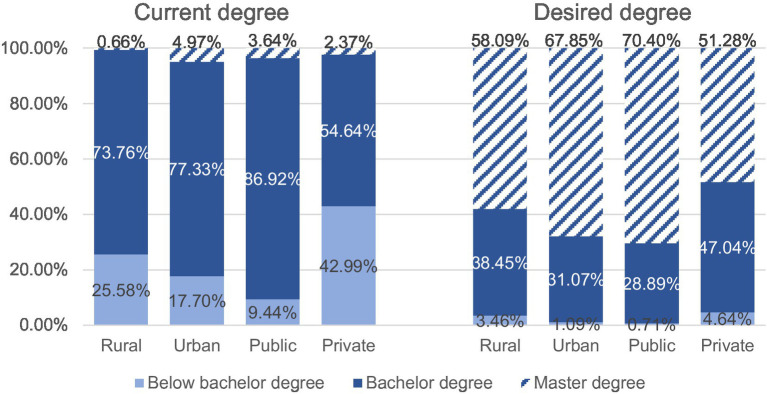
Current and desired degrees of different kindergarten principals (*n* = 2,963).

As shown in Figure 2, most public participants (90.56%) had a bachelor’s or master’s degree. Nearly half of the private participants had a college degree or less. Over 70% of public participants desired a master’s degree in the future, compared to half (51.28%) of private principals. Nearly half of the private principals (44.08%) desired a bachelor’s degree, while 86.92% of public principals already had bachelor’s degrees.

Generally, the public-private degree gaps (current degree: *F* = 400.829, *p < 0.001*; desired degree: *F* = 130.735, *p < 0.001*) were more distinct than those between rural–urban participants (current degree: *F* = 63.554, *p* < 0.001; desired degree: *F* = 39.630, *p* < 0.001).

### 4.2. PD needs and preferred program contents

A two-way between-groups ANOVA was conducted to compare the PD needs of rural–urban and public-private participants, and the results are presented in Table 3. The two most frequently selected PD needs were ‘To improve leadership’ (*M* = 4.844 ± 0.50) and ‘To improve expertise’ (*M* = 4.813 ± 0.54). There were significant urban–rural differences in the needs ‘To obtain a higher degree’ (rural = 4.18; urban = 4.00; *p <* 0.001) and ‘To get a certificate’ (rural = 4.29; urban = 4.21; *p* < 0.05). Significant differences between public-private participants were found for most items. The public participants had stronger needs for the first five items but less interest in the last three items (‘To improve marketing skills’, ‘To obtain a higher degree’, and ‘To get a certificate’) than private principals.

**Table 3:** Two-way ANOVA: PD needs and preferred PD program contents.

Factor	Indicator/Item	Public-Private	Urban–Rural	Public-Private * Urban–Rural		Urban	Rural
		*F*	*F*	*F*		M ± SD	M ± SD
PD needs	To improve expertise	22.927**	3.743	2.709	Public	4.85 ± 0.47	4.84 ± 0.50
					Private	4.78 ± 0.59	4.70 ± 0.69
	To improve leadership	21.897**	5.586*	0.045	Public	4.89 ± 0.38	4.85 ± 0.52
					Private	4.80 ± 0.55	4.75 ± 0.63
	To learn ECE content	19.395**	8.423**	7.779**	Public	4.82 ± 0.52	4.81 ± 0.54
					Private	4.78 ± 0.60	4.65 ± 0.75
	To meet researchers	38.647**	6.876**	1.585	Public	4.79 ± 0.53	4.75 ± 0.62
					Private	4.66 ± 0.72	4.56 ± 0.79
	To meet other principals	6.199*	3.128	6.492*	Public	4.63 ± 0.71	4.65 ± 0.71
					Private	4.63 ± 0.72	4.50 ± 0.83
	To improve marketing skills	3.379	5.744*	7.923**	Public	4.48 ± 0.90	4.50 ± 0.94
					Private	4.65 ± 0.76	4.46 ± 0.91
	To obtain a higher degree	16.846**	13.891**	1.275	Public	3.90 ± 1.36	4.14 ± 1.22
					Private	4.16 ± 1.22	4.29 ± 1.06
	To get a certificate	12.211**	2.556	3.917*	Public	4.11 ± 1.18	4.27 ± 1.10
					Private	4.36 ± 1.10	4.34 ± 1.04
Preferred PD program contents	Planning the development of kindergarten	13.356**	0.954	3.252	Public	4.21 ± 0.77	4.23 ± 0.76
					Private	4.15 ± 0.81	4.06 ± 0.81
	Creating educational culture	38.678**	0.314	2.276	Public	4.42 ± 0.85	4.46 ± 0.80
					Private	4.25 ± 1.01	4.18 ± 1.01
	Leading care and education	69.844**	2.363	5.699*	Public	4.42 ± 0.65	4.45 ± 0.66
					Private	4.25 ± 0.77	4.14 ± 0.85
	Guiding teachers’ development	5.639*	0.880	3.304	Public	4.39 ± 0.74	4.41 ± 0.72
					Private	4.37 ± 0.77	4.29 ± 0.78
	Improving management	8.211**	0.240	2.551	Public	4.34 ± 0.73	4.37 ± 0.71
					Private	4.30 ± 0.73	4.24 ± 0.78
	Adapting to the environment	7.543**	0.509	15.370**	Public	3.92 ± 0.95	4.09 ± 0.91
					Private	4.17 ± 0.86	4.05 ± 0.95

*p < 0.05, **p < 0.01.

The most needed PD program content was ‘Guiding teachers’ development’ (*N* = 2,713, 91.56%). There were no significant rural–urban and public-private differences in this item. The public participants had stronger needs in the first five content items, but private principals indicated a stronger need for the last item, ‘Adapting to the environment’.

The two-way ANOVA revealed a significant interaction effect between rural–urban and public-private participants. The interaction effect between the four groups of kindergarten participants was statistically significant in the following items: ‘To learn ECE content’ (*F* = 7.779, *p* < 0.005), ‘To meet other principals’ (*F* = 6.492, *p* < 0.05), ‘To improve marketing skills’ (*F* = 7.923, *p* < 0.005), ‘To get a certificate’ (*F* = 3.917, *p* < 0.05), ‘Leading care and education’ (*F* = 5.699, *p* < 0.05), and ‘Adapting to the environment’ (*F* = 15.370, *p* < 0.001). In other items, the interaction effect was not statistically significant, and the effect size was small, *η_p_^2^* (public-private) = 0.023; *η_p_^2^* (rural–urban) = 0.005, *η_p_^2^* (rural–urban * public-private) = 0.005.

### 4.3. Three profiles of PD needs

We conducted an LPA to explore participants’ PD needs based on the two dimensions in Table 3, ‘PD needs’ and ‘preferred PD program contents’; it yielded five models with varying numbers of latent classes. As shown in Table 4, the three-class solution was demonstrated to have the best model fit. The values of the Akaike information criterion (AIC), Bayesian information criterion (BIC), and adjusted BIC (aBIC) decrease continuously as the number of classes increases. As the number of classes changes from three to four, the values of AIC, BIC, and aBIC demonstrate a relatively slight change. Table 4 presents this three-profile model, which was selected because it had the highest entropy (0.92), lower AIC (8,499.489) and BIC values (8,559.428), a slightly lower aBIC value (8527.654), and a statistically significant LMRT value (*p* < 0.001). The parsimony and interpretability of the three profiles were also considered. Based on the results of all model fitting indexes, the three-profile model was supposed to be a perfect model.

**Table 4:** Latent profile analysis: model-fit statistics of the potential models (*n* = 2,963).

Model	AIC	BIC	Adjusted BIC	*p*-LMR	Entropy	Percentage in profiles
C = 2	9,440.148	9,482.106	9,459.864	0.0000	0.886	0.195/0.805
**C = 3**	**8,499.489**	**8,559.428**	**8,527.654**	**0.0001**	**0.920**	**0.075/0.222/0.703**
C = 4	8,122.724	8,200.645	8,159.339	0.1211	0.917	0.043/0.092/0.652/0.213
C = 5	7,683.837	7,779.740	7,728.902	0.1662	0.930	0.036/0.059/0.587/0.127/0.191

AIC, Akaike information criterion; BIC, Bayesian information criterion; p-LMR, the value of *p* of the Lo–Mendell–Rubin likelihood ratio test; Bold values indicate the most suitable model (three-profile model) based on the results of all model fitting indexes.

**Figure 3:**
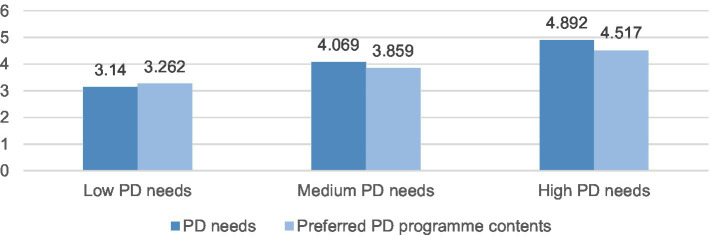
Mean scores of PD needs and preferred PD program contents across three profiles.

As shown in Figure 3, 7.5% (*n* = 222) of participants were classified into Profile 1, 22.2% (*n* = 657) as Profile 2, and 70.3% (*n* = 2,084) as Profile 3. First, Profile 1 was labeled ‘Low PD needs’ as they had the lowest scores on eight ‘PD needs’ items (*M* = 3.140 ± 0.35) and six ‘preferred PD program contents’ items (*M* = 3.262 ± 0.558). Second, Profile 2 was labeled ‘Medium PD needs’ (PD needs: *M* = 4.069 ± 0.242; preferred PD program contents: *M* = 3.859 ± 0.561). Finally, Profile 3 was labeled ‘High PD needs’ (PD needs: *M* = 4.892 ± 0.173; preferred PD program contents: *M* = 4.517 ± 0.569).

A chi-square analysis was conducted to investigate the differences among the demographic variables of the three profiles. As shown in Table 5, the three profiles were differentiated by age (*p* < 0.01), years of administrative work experience (*p* < 0.01), current degree (*p* < 0.05), desired degree (*p* < 0.01), years since their kindergarten was established (*p* < 0.01), and whether they worked in public or private kindergartens (*p* < 0.01). No significant differences were found between rural–urban participants.

**Table 5:** Demographic characteristics according to the three profiles.

Variable	Profile1	Profile2	Profile3	χ^2^	*P*	Cramer’s V
	n = 222	n = 657	n = 2084			
Age				142.998	0.000**	0.155
20–30	6.18%	13.48%	80.34%			
31–40	5.40%	17.42%	77.19%			
41–50	8.47%	26.10%	65.43%			
≥ 51	15.86%	41.41%	42.73%			
Years of administrative experience				69.072	0.000**	0.108
≤3	5.34%	17.67%	76.98%			
4–7	8.82%	20.39%	70.80%			
8–10	6.88%	28.44%	64.68%			
11–15	7.47%	27.27%	65.26%			
≥16	12.91%	28.83%	58.26%			
Current degree				20.005	0.010*	0.058
Master’s degree	9.47%	28.42%	62.11%			
Bachelor’s degree	6.54%	22.55%	70.91%			
College degree	10.18%	20.00%	69.82%			
Teacher’s secondary school	12.77%	21.28%	65.96%			
High school	23.08%	7.69%	69.23%			
Desired degree				100.136	0.000**	0.130
Master’s degree	4.70%	19.45%	75.85%			
Bachelor’s degree	11.98%	27.33%	60.69%			
College degree	20.00%	21.82%	58.18%			
Teacher’s secondary school	16.67%	16.67%	66.67%			
Number of years kindergarten has been established				28.389	0.000**	0.069
≤3	4.06%	19.40%	76.54%			
4–7	6.99%	21.68%	71.33%			
8–12	9.03%	22.65%	68.32%			
13–20	8.30%	19.29%	72.41%			
≥21	8.72%	25.92%	65.36%			
Urban or rural				2.238	0.327	0.027
Urban	7.08%	22.96%	69.96%			
Rural	8.09%	21.04%	70.87%			
Public or private				14.778	0.001**	0.071
Public	6.57%	23.91%	69.52%			
Private	9.27%	18.84%	71.89%			

*p < 0.05, **p < 0.01.

#### 4.3.1. Low PD needs profile

Participants in Profile 1 had the lowest scores on these items; this group was named the “Low PD needs” group, with the least number of participants; only 222 (7.5%) principals belonged to this group. The major features of the low PD needs group were: being over 50 years old (15.86%), having more than 15 years of administrative experience (12.91%), having a high school degree (23.08%), working in private schools (9.27%), and working at schools established for 8–12 years (9.03%). This result shows that experienced and relatively senior private principals with high degrees working at kindergartens established for 8–12 years are more likely to have low PD needs.

#### 4.3.2. Medium PD needs profile

The major features of the medium profile group were as follows: being over 50 years old (41.41%), having over 15 years of administrative work experience (28.83%), having a master’s degree (28.42%), working in a public school (23.91%), and working at schools established for 8–12 years (22.65%). This result indicates that veteran public kindergarten principals with master’s degrees over 50 years old and working at kindergartens founded 8–12 years ago tend to have medium PD needs.

#### 4.3.3. High PD needs profile

The major features of the high PD needs group included: being in the 20–30 years age group (80.34%), having less than 3 years of administrative experience (76.98%), having a bachelor’s degree (70.91%), desiring a master’s degree (75.85%), working in a private school (71.89%), and working at schools established for less than 3 years (76.54%). This result indicates that novice principals at new schools with bachelor’s degrees and desiring master’s degrees are likelier to have high PD needs.

### 4.4. Obstacles to PD

As shown in Table 6, the ANOVA test revealed significant differences between rural–urban and public-private principals regarding the obstacles to their PD. The public principals reported more severe concerns about PD program content and training style (*M* = 3.85 and *M* = 3.87, respectively) than those in private schools (*M* = 3.71 and *M* = 3.64, respectively).

**Table 6:** Two-way ANOVA: obstacles to PD.

Title	Public/Private*Urban/Rural	Public	Private	*F*	Urban	Rural	*F*
	*F*	M + SD	M + SD		M + SD	M + SD	
PD content is too theoretical	1.480	3.85 ± 1.07	3.71 ± 1.17	12.67**	3.81 ± 1.11	3.79 ± 1.10	1.64
Boring PD style	0.561	3.87 ± 1.15	3.64 ± 1.28	24.83**	3.84 ± 1.21	3.71 ± 1.19	9.63**
Inconvenient PD time	1.580	3.55 ± 1.15	3.47 ± 1.17	5.91*	3.58 ± 1.14	3.45 ± 1.18	12.839**
Inconvenient PD location	0.007	3.37 ± 1.18	3.35 ± 1.17	0.33	3.40 ± 1.17	3.31 ± 1.19	4.411*

*p < 0.05, **p < 0.01.

Both public and urban principals reported higher concerns about PD training style than those in private and rural schools (public = 3.87; urban = 3.84; private = 3.64; rural = 3.71). Generally, urban principals valued their time and cared more about the training styles and locations of PD than principals in rural areas. However, the interaction effect between the kindergarten type and the region was not statistically significant among the four items.

### 4.5. Interviews: the 3A problems in principals’ PD

To understand other factors that facilitate or hinder principals’ PD needs, we analyzed in-depth interviews using the 3A framework based on accessibility, affordability, and accountability perspectives.

#### 4.5.1. Accessibility

We defined ‘accessibility’ based on whether principals could easily access PD opportunities. All urban public participants stated that they could easily attend PD programs twice a year, while rural public participants only attended once or twice yearly. However, seven of the eight private participants felt it was hard to attend the PD program even once a year, especially those in rural private schools. In addition, participants F and N bluntly described in-service PD opportunities as ‘non-existent’. The following statements support this:

*“Three years ago, I attended a PD session to get a principal certificate as the policy required. Since then, I have never had any opportunities for PD. There were many PD opportunities for public principals but nothing for us.*” *(F, urban private principal).*

*“I do need to improve my expertise, management, and degree. I faced so many challenges. I only have a college degree. Oh! Half of the teachers have higher degrees than me. But I did not hear about any PD opportunities this year.” (N, rural private principal).*

In summary, the public-private gap in accessibility was remarkable. Most private principals could not attend appropriate and conveniently located PD programs, though they felt a strong need to improve their degrees, leadership, or expertise. Some participants were stressed, anxious, and even embarrassed about not having appropriate opportunities for PD.

#### 4.5.2. Affordability

Affordability was defined as whether principals could easily afford the fees and time involved in pursuing PD programs. Most PD programs provided by local authorities were free for public principals. Even public principals in rural areas stated that their fees for PD programs could be reimbursed. However, half of the public participants complained that they could hardly find the time for PD programs. Their high workload contributed to a tendency among principals to avoid in-service PD opportunities, as confirmed by principal A:

*“I do not have time for PD programs. Too much workload! I prefer short-term programs, like one or two days in the summer break. The national Key Principal program lasts for one month! How can I find 30 days to leave my position? It’s hard for me to find half a day away.” (A, urban public principal).*

While the public participants complained about their busy schedules, private participants believed they had neither time nor funds for PD. For example, M, a rural private principal, stated:

*“My training will not be reimbursable like those in public schools. If my boss asked me to attend the PD program, she might cover the fee, but she assumes I do not need any training. Besides, I am too busy.”*

Thus, the affordability gap between public and private participants was also significant. All the private participants expressed their hope for funds or bursaries for further PD and stated that they desperately need to improve their leadership and expertise.

#### 4.5.3. Accountability

Accountability refers to how the PD program should be accountable for improving the principal’s professionalism. For example, although some participants (A and D) indicated that they found the PD program to be fruitful, other participants complained about the quality of previous PD programs, in which lectures or presentations were used to transmit theoretical knowledge; participants were treated as passive recipients of ready-made knowledge rather than active agents, as the following statement shows:

*“I would suggest a more practical PD program for us in rural areas. It seems like all the PD programs are designed for urban principals. It is not practical for my school. I’d like to visit other rural kindergartens instead of listening to a talk.” (L, Rural public principal).*

*“Well, it was boring. The trainers often lacked management experience and taught some theories which seemed useless to us. The trainer gave us some evaluation forms. But I do not think it’ll work. I have attended many PD programs. Most of them were the same. Someone [in the government] should monitor the PD programs.” (B, Urban public principal).*

In summary, most respondents were dissatisfied with the current quality of PD programs. The survey results verified this finding. Most participants suggested that there should be a more consistent evaluation rubric for PD programs and that the government should monitor the effectiveness of PD programs.

## 5. Discussion

This first national survey of the PD needs of Chinese kindergarten principals has confirmed the rural–urban and private-public gaps, verified by the follow-up interview studies. This section will discuss these findings and their implications for PD policymaking and practical improvements.

### 5.1. The rural–urban gap in PD needs

This study found a significant rural–urban gap in PD needs among Chinese kindergarten principals. This is supplementary to a previous study suggesting that principals in rural areas may have specific PD needs (Salazar, 2007). In particular, this study found that more rural principals were novices within the first three years of their leadership position (Shoho and Barnett, 2010). Due to the rapid expansion of ECE, the number of kindergartens in China climbed by 94%, from 150,400 in 2010 to 291,700 in 2021 (Gov. CHN, 2011–2022). These new kindergartens were mainly located in rural areas, and thus, more novice principals emerged in rural kindergartens. Accordingly, the novice principals in this study reported strong PD needs. This finding is consistent with the existing findings: novice principals experience more challenges in building and sustaining community relationships and thus need more PD training (Bloom, 1989; Hargreaves, 2005; Meyer and Patuawa, 2022).

In addition, this study found a significant difference between rural and urban participants’ educational levels. More urban participants had bachelor’s and master’s degrees than those in rural areas; in particular, the urban–rural ratio of master’s degrees was 8:1. This widening urban–rural gap in educational levels has made rural principals (the disadvantaged group) need PD training more. As a growing body of research suggests that principals’ educational level is a strong predictor of overall ECE quality (Helburn et al., 1995; Bloom, 2004; Vu et al., 2008), it is expected that the quality of rural kindergartens is far behind their urban counterparts (Li et al., 2019; Qian, 2019). Therefore, it is urgently needed to provide more PD opportunities for rural principals and to upgrade their educational levels to promote educational equity in China.

### 5.2. The public-private gap in PD needs

This study found a significant public-private gap in the PD needs of Chinese kindergarten principals. In addition, this study also found that the public-private degree gap was larger than the rural–urban gap. Half the private kindergarten principals had not completed a bachelor’s degree, and the principals in rural private kindergartens had the lowest educational level and became the most disadvantaged group. This finding is consistent with a previous study (Yin, 2022). In addition, private kindergartens in rural China have a limited budget for extra expenditures such as PD programs. Although the *Outline* targeted increasing government responsibilities in supporting private kindergartens, private principals struggled to get appropriate PD opportunities, which would have enlarged the public-private gap.

### 5.3. The ‘Matthew effect’ in Chinese kindergarten principals’ PD

This study found that more than 70% of principals had high PD needs, and most of them were novice principals in new private kindergartens in rural China. Unfortunately, they had few resources and opportunities for PD under the current ECE structure in China: private-public dichotomy and rural–urban divide. In contrast, those principals in urban public kindergartens had more PD opportunities even though they did not need them. These findings jointly indicated a ‘Matthew Effect’ in Chinese principal PD: the rich would get more, whereas the poor would get less. This ‘Matthew Effect’ implies that the current PD policy in China is dysfunctional or ineffective in narrowing the urban–rural and public-private gaps; instead, the policy has enlarged the gaps and resulted in the current challenges that have limited the offer of free PD programs to all principals. Previous studies have reported that very few novice and private principals could participate in the national principal PD program (Xing and Yang, 2018). When their ‘sisters’ (the principals of urban public kindergartens) attend the ‘ball’ (PD training) organized by the ‘prince’ (the educational authorities), ‘Cinderella’ (the principals of rural private kindergartens) is crying in the kitchen. However, no policy amendments have been made, and the bureaucratic policymakers continue to train the most experienced and effective principals, leaving ‘Cinderellas’ still crying. Suppose this tragedy continues; thus, the rural and private principals with high PD needs cannot access PD programs. In that case, the public-private and urban–rural gaps in kindergartens will continue to widen. The policymakers should act now to stop this ‘Matthew Effect.”

### 5.4. Limitations

This is the first national survey to evaluate kindergarten principals’ PD needs in China. However, this study has three major limitations that should be addressed in future studies. First, this study adopted the cross-sectional design that surveyed Chinese principals at one study time point. This design does not allow us to precisely measure the changes in principals’ PD needs, especially between the time points before and after the PD training. Future studies should preferably consider longitudinal studies to deepen the understanding of Chinese principals’ evolving changes in PD needs. Second, this study employed a newly developed questionnaire asking Chinese principals to self-report their PD needs. This self-reported survey is vulnerable to socially desirable bias, which is the respondents’ tendency to over-report their needs and thus interferes with the interpretation of average tendencies and individual differences. To overcome the bias, future studies should establish a triangulation of methods such as surveys, interviews, document analysis, and field notes. Third, this study only surveyed Chinese principals about their PD needs, leaving other stakeholders such as teachers, parents, and education officers uninvolved. This single-informant study is likely to suffer from an informant bias, which negatively impacts the validity of the findings. Future studies should involve early childhood teachers, parents, and educational officers to provide triangulated perspectives and consolidated evidence.

### 5.5. Implications

The findings of this study have some implications for policymaking: (1) Accessibility: all principals, whether public or private, rural or urban, should have equal access to PD programs; (2) Affordability: free PD programs should be provided to those rural and private principals; and (3) Accountability: the PD programs should be improved to enhance their efficiency and attractiveness. Accordingly, more efforts should be made to solve the ‘3A’ problems facing Chinese principals’ PD, along with increasing the fiscal budget in the private PD sector and monitoring the quality of PD. This will promote the sustainability and social justice of ECE. Furthermore, integrated policies and efficient PD programs should be developed and tailored to the PD needs of different kindergarten principals. The educational authorities should act now to ensure that all principals achieve high-quality PD and stop the ‘Matthew effect’ in the PD and the entire ECE field.

## Data availability statement

The raw data supporting the conclusions of this article will be made available by the authors, without undue reservation.

## Ethics statement

The studies involving human participants were reviewed and approved by East China Normal University Ethics Review Committee (HR004-2021). The patients/participants provided their written informed consent to participate in this study.

## Author contributions

YQ: methodology, writing—original draft preparation, and resources. SZ: investigation and data curation. HL: conceptualization, writing—review and editing, and supervision. All authors contributed to the article and approved the submitted version.

## Funding

This research was funded by the Program of Humanities and Social Sciences, Ministry of Education, China, funding number 21YJAZH066; and Shanghai Educational Legislative Talent Program, funding number 2020JYFXR040.

## Conflict of interest

The authors declare that the research was conducted in the absence of any commercial or financial relationships that could be construed as a potential conflict of interest.

## Publisher’s note

All claims expressed in this article are solely those of the authors and do not necessarily represent those of their affiliated organizations, or those of the publisher, the editors and the reviewers. Any product that may be evaluated in this article, or claim that may be made by its manufacturer, is not guaranteed or endorsed by the publisher.
